# Engagement and social acceptance in genome editing for human benefit: Reflections on research and practice in a global context

**DOI:** 10.12688/wellcomeopenres.16260.2

**Published:** 2021-07-02

**Authors:** Sebastián Barbosa, Lea Pare Toe, Delphine Thizy, Manjulika Vaz, Lucy Carter

**Affiliations:** 1 Ministry of Science, Technology and Innovation, Buenos Aires, Argentina; 2 Institut de Recherche en Sciences de la Santé, Ouagadougou, Burkina Faso; 3 Imperial College London, London, UK; 4 St John’s Research Institute, St John's Medical College, Bengaluru, India; 5 CSIRO Land and Water, Brisbane, Australia

**Keywords:** Public engagement, gene drives, genome editing, social acceptance

## Abstract

While there are both practical and ethical reasons for public engagement in science and innovation, real-world detailed examples of engagement practice and the lessons to come from these are still hard to find. This paper showcases three contextually diverse case studies of engagement practice. Case 1 recounts the experiences of a government-funded initiative to involve scientists and policy makers as science communicators for the purpose of engaging the Argentine public on gene editing. Case 2 describes the research methodologies used to elicit diverse stakeholder views in the face of political uncertainty and institutional distrust in India. Finally, case 3 unpacks the tensions and gaps with existing international guidelines for ensuring local voices are respected in community decision-making in Burkina Faso. Each case shares its own compelling rationale for selecting the engagement method chosen and details the challenges encountered along the way. Each case shares its vision for creating legitimate opportunities for broader societal involvement in the planning, conduct and delivery of responsible science. These cases demonstrate the nuances, sensitivities and challenges of engaging with publics and broader stakeholders in discussions about genome editing for human benefit.

## Disclaimer

The views expressed in this article are those of the authors. Publication in Wellcome Open Research does not imply endorsement by Wellcome.

## Background

Decades of scholarship from social and interdisciplinary sciences, and the humanities, have attempted to conceptualize, theorize and defend the principles and practices considered essential for involving various publics in the governance of innovation ( McNoughton (2012); NASEM, 2016; Rowe & Frewer, 2005; Stirling, 2008; Wynne, 2001). As more empirical studies begin to emerge which include reflections on engagement experience, important insights about the *practice* of engagement are shedding light on the challenges, resources, and motivations that make engagement initiatives both successful and publicly acceptable ( Burgess *et al*., 2018; Kokotovich *et al*., 2020). There are a few key lessons from this body of work and several of these are explored in the case studies presented here. Some of them include: the multiple and at times conflicting values, interests and knowledges at play in the development and use of scientific innovations; the varying rationales for involving publics and communities in the delivery of science outcomes and; the intensive planning, commitment and resourcing that quality engagement and communication initiatives demand. The manner in which engagement is conceived, planned, funded and implemented, that is, the way engagement is governed, is the focus of this collection of engagement stories.

The case studies presented here illustrate the diverse contexts in which engagement occurs and the varying roles engagement scientists and practitioners can assume. Each case is set in a specific country context where public and stakeholder involvement in science has different histories, where regulation and governance are at different stages of maturity, and where the drivers for public involvement diverge. The case studies presented are unique in a number of ways. They span diverse global regions to include a large public communications event in Argentina (Case study 1), the use of social research methodologies to engage with diverse publics in India (Case study 2) and a co-development and engagement model for involved communities in Burkina Faso (Case study 3). The cases also, to varying extent, cover a broad spectrum of engagement originally identified by Rowe & Frewer (2005) which includes public communication, public consultation and public engagement.

The cases shared highlight layers of historical and institutional tensions between research and practice, between the differing roles of organisations and their mandates, and the burden of demonstrating legitimacy in the funding and implementation of responsible science and innovation ( Kokotovich *et al*., 2020). This collection shares valuable and practical information and insights into ethical engagement *practice* where aspirations of ethical public engagement meets the realities of achieving transparent, inclusive and legitimate forums for interaction.

## Case 1: Raising public awareness of gene editing in Argentina

In recent times, Argentina has invested heavily in molecular biology science and technology development, enabling a solid base from which to advance the development of gene editing (GE) regionally. In November 2018, Argentina declared its support for GE to the World Trade Organization, demonstrating its strong national support and consensus for continuing research and development in genetics ^1^. Given this setting, and with the intention of providing the general public with reliable information on the country’s position, a partnership of Argentine government organisations co-convened the first public outreach event in Argentina to raise awareness of GE technologies and their benefits.

In December 2018, a group of partners funded by Argentine government organisations came together to decide upon the information to be communicated and the most suitable style of communication to achieve the objective of providing clear and reliable information about GE. The group was comprised of the Ministry of Science, the Ministry of Health, the Ministry of Agroindustry and three partnering organisations: The National Council of Scientific and Technical Research, the National Institute of Agricultural Technology, and the National Administration of Laboratories and Institutes of Health.

The primary objective of this event was to engage the public on the status of GE in Argentina.

The event was transmitted via streaming and social media networks, mostly Twitter ^2^. Being co-organized by six separate government entities, the communication focused on multiple GE contexts including health research, food technology and agriculture - three contextual applications most likely to attract research interest and progress in the area of CRISPR technology ^3^. The primary resources allocated to this event included covering the salaries of organizers and technical operators, provision of travel reimbursements for speakers, rental of technical equipment and catering for attendees.

In total, nine invited speakers, many of them researchers already working with CRISPR in health research, agriculture, and food technology sectors, along with decision makers in policy matters were invited to speak at the event. The information presented was targeted for the general public and presenters were coached in communicating effectively to a general audience. The event attracted 452 attendees, while 196 participants viewed the event using live streaming platforms and the tweets generated from the Twitter account of the Ministry of Science and Technology (MINCyT) received over 40,000 impressions. The event attracted broad interest in the mainstream media, with 19 news stories written in national newspapers and online-news websites two days after the event ^4^. Ongoing coverage also included the participation of journalists and non-for-profit foundations concerned with health and food technology who tweeted during the event, helping to increase the impact of the messages delivered.

The challenges of designing and organizing an event of this scale were multiple. They included: challenges relating to the content and delivery of scientific information, taking care to build public trust in the science and governance of GE technologies, and fostering co-operation and collaboration among stakeholders and organizers in presenting a unified message.

In relation to science communication, the goal of the initiative was to enable the audience to participate in discussions about science by providing concise and, reliable information that was publicly accessible. A communication strategy was developed to define the content and most suitable style of communication to achieve the event’s objectives. Technical terms were replaced by simple concepts in a language easily understood, focusing on the benefits of GE but also mentioning aspects for improvement. Text was replaced by images for all presenters acknowledging the power of images over text for generating human understanding. Each speaker was coached by a communication expert in the delivery of effective presentations.

Based on previous public engagement lessons with the introduction of genetically modified organisms in the early 2000s ( Rhodes & Sawyer, 2015), organizers used the event to decisively build public trust in regulatory oversight and governance processes. Effort was made to inform the public about GE before any gene edited products could reach the market. The provision of accurate and transparent information on the current status of gene edited products under development for agricultural use, food production and human health was a key feature of presentations. Effort was also made to explain the regulatory and bioethical frameworks in place in Argentina since 2015 and that oversight review committees have prior to product approval. Finally, organizers worked hard to avoid creating false expectations of GE as a panacea for solving complex problems.

Working collectively allowed organizers to present a strong message, allowing for deeper impact and demonstrating cooperation in governance of the issues. It was important that such initiatives did not occur in isolation but informed and involved stakeholders more broadly. A key goal was to include as many stakeholders and participants during and after the event as possible.

Question and answer sessions were included at the end of each panel in order to engage the public in the debate. These sessions allowed organizers to identify the publics’ concerns regarding the emerging technologies introduced. Questions from the audience revealed a good understanding of the information presented in the presentations and demonstrated interest in the future potential of GE. Following a presentation of ongoing clinical trials with GE products, the public showed interest in the timeframes for therapies becoming available and whether they would be safe for human use. After presenting information on the local development of a GE potato, the public expressed interest in knowing whether the product could be exported and whether the government expected to generate revenue for Argentina from this innovation.

Organizers and presenters were committed to resisting making promises which could potentially diminish trust in the message delivered. Responses to questions about safety and efficacy were met with messages about the importance of robust regulatory and governance processes and that every product or therapy was to be evaluated as the technology develops. In relation to whether the therapies and products were safe, event organizers assured the public that regulatory agencies were assessing each development on a case-by-case basis.

The event was positively received by attendees (as indicated by the social media activity following the event) and attracted wide media coverage. Since the event, two speakers have been invited on two occasions to speak on radio after the host read about the event on Twitter and organizers have since received invitations to convene two additional events on the topic of GE, aimed to engage with medical doctors ^5^.

## Reflections on Case 1

The challenges and lessons in organizing and delivering a large-scale public event designed to communicate information about the status of GE in Argentina to a diverse audience were multiple. For organizers, the experience was both challenging and exciting in that they were tasked with delivering an event without precedence and without previous experience to draw upon. Never before had topics relating to GE been directly communicated to the general public by multiple government institutions. While this situation presented a great opportunity to shape the event, it also presented a challenge given that no previous comparable events had been held to fully guide organizers.

An important reflection for organizers was a possible missed opportunity to carry out a post-event communication strategy to continue informing the public and to help consolidate the issues raised. Profiles could have been created on social media that continued to report new developments introduced during the event and on other topics related to GE occurring nationally and globally.

Paying attention to communication design aspects which retained accountability, trust and transparency had served organizers well. In reflecting on these experiences, we offer the following recommendations for others attempting to convene similar events, each of which we consider essential:

➢ Communication methods must be tailored to the audience you intend to reach. It is important to define communication objectives, focus content, and determine the best mechanisms of communication. Nothing should be left to chance.
➢ CRISPR technology should not be communicated as a ‘super’ technique that has the potential to make rapid impact on every aspect of society. This will undermine public perceptions and trust in future messaging.
➢ Resist making comparisons between CRISPR technology and other genomic techniques to promote the benefits of CRISPR. Each technology and application have a set purpose, along with distinct benefits and disadvantages. Communicating truthfully but simply has proved to be successful for event organizers.

## Case 2: Eliciting public views and promoting dialogue using social science in India

While engagement research has great value in the development of ethical and human-centred policy for technology development, there have been limited in-depth empirical studies to understand lay perspectives in relation to genetics, genomic research and genetic technologies. This is especially true for India. As a culturally pluralistic country, India has diverse belief systems and religious traditions which influence people’s worldviews and practices. In contemporary times, nationalist sentiments and a push for speedy scientific advancement appear to have spurred the Indian government to formulate and present to Parliament, a *DNA Technologies Bill* (2019) and *the National Guidelines for Gene Therapy Product Development & Clinical Trials,* 2019 ( ICMR *et al*., 2019). In early 2020, a draft document on *Genome Edited Organisms: Regulatory Framework and Guidelines for Risk Assessment* was formulated by the Department of Biotechnology, Ministry of Science & Technology ( DBT, 2020) which takes into consideration the biosafety and security aspects of genome editing technologies but contains no mention of ethical or social considerations. The 2017 *Ethical Guidelines* by the Indian Council of Medical Research ( ICMR, 2019) allows use of somatic cells for therapy, including gene therapy with some conditions subject to Rules 1989 of the *Environment Protection Act* 1986.

Despite these legal instruments, there remains uncertainty about India’s regulatory capacity to enforce ethical standards for GE and CRISPR use ( Udwadia & Singh, 2019). India has previously experienced the circumventing of regulation by unscrupulous individuals’ intent on achieving personal and institutional gains. This occurred with genetically modified (GM) crops where prior to final sanctions in parliament, GM cotton was sown in Gujarat, creating more fear than confidence in the technology and its regulatory processes ( Udwadia & Singh, 2019). It is with this backdrop that national discussions are now taking place in India. There is no better time to hold open discussions that are responsive to the concerns and expectations of citizens that are those most likely to be affected by the technologies’ promises.

Earlier work of Vaz *et al*. has shown that health literacy in India is low, not just among the least educated but among publics in general ( O'Doherty *et al*., 2012; Vaz *et al*., 2015). However, in recent years public mistrust of the health system and the assumed exploitative nexus between research and industry has left people cynical and skeptical, with distrust towards authorities commonplace. Concurrently, mainstream and social media has provided people with information about medical advancements, successful novel treatments and sensational, novel experiments including the Chinese case of GE of human embryos for HIV prevention ( Rana, 2019). In India, journalists have appealed to regulators and scientists for more dialogue with lay publics on the ethics of GE and its “grey areas of technical and socio-political implications” ( Padma, 2018). Adding to this mix of public commentary, social activists and non-government organisations are active and vocal on the introduction of new schemes, projects and sociopolitical and ethical issues.

This case defined public engagement as a dynamic, evolving process which challenged the notion of clear boundaries between ‘expert knowledge’ and ‘lay knowledge’ ( Goisauf & Durnová, 2019). In some situations, engagement is limited to the dissemination of expert knowledge but in the early stages of introducing new technologies, understanding public perceptions, expectations and concerns and the beliefs, customs and life experiences that underpin these, is critical for achieving some level of public acceptance and trust. The creation of people-centred regulatory and governance frameworks which ensure scientists and researchers are aware and responsive to the sentiments of the public who stand to benefit from such research is a critical step in the process of creating the trust necessary for acceptance ( Molster *et al*., 2012; Walmsley, 2009). Deliberative dialogue can be a useful mechanism for community engagement, especially where democratic and participatory practices are valued ( Tindana *et al*., 2017).

Engagement with communities helps to enhance people’s understanding of the methods and tools of genomic research and provides an opportunity to negotiate boundaries where arrival at a shared understanding is possible. Well-executed engagement processes have the potential to balance biomedical conceptions of illness with traditional culturally-held beliefs about illness and health ( Tindana *et al*., 2017). Engagement can also shed light on diverse values arising from religious beliefs, cultural norms and community traditions and improve the research community’s understanding of existing public fears, mistrust and miscommunication. It is also important that the goals of science are aligned with that of society.

In a qualitative study conducted in Bengaluru in 2014–2015, the perceptions of the lay public, the research community and members of various ethics committees were explored to help elicit views on the ethics of biobanking research ( Vaz *et al*., 2015). Using an unfolding case vignette ^6^, useful insights emerged for how the research community perceived and conducted engagement with others. While members of the general public held broad concerns ranging from fears about misuse, eugenics, the prospect of manipulating nature, and the risk of commercial exploitation; on the whole, they were positive about the possibilities of genetic research benefiting society and helping their children prevent future disease. The public indicated a wish to be informed that such research was underway, particularly to ensure that the researcher was held accountable and that they themselves had a voice. In the words of one participant, “ *They should inform us if they are doing genetic research… it is not about us understanding or objecting… but making them know that we matter*”.

Building on these findings our research team presents a follow-on study in Bengaluru, to explore people-centred governance mechanisms for the use of stored samples for genetic research ^7^. A person-centred-approach here refers to providing the enabling conditions which support active involvement of participants in decision-making about issues that affect them. Both studies offer insights for how the *process* as well as the *outcomes* of using social science methodologies can assist in our understanding of public attitudes towards scientific advances aiming to use genetic information and techniques for human benefit.

Fundamental to our approach in this second study was the recognition that the public is not a fixed body of individuals. It is composed of persons interested in an issue, who can affect research outcomes by supporting or opposing the other actors involved ( Gottweis *et al*., 2011). Instead of immediately engaging individual stakeholders, we chose to facilitate a deliberation among a heterogenous group of members with praxis expertise (i.e. those with interest in people-centred advocacy and governance mechanisms in their respective fields). This approach allowed for participants from a range of backgrounds and experiences to come together to identify shared values and common concerns. The group had expertise in citizen action, data management, patient advocacy and health policy advocacy. To ensure that participants had a baseline understanding of the subject for the purposes of deliberation, an information packet with background reading material was provided. Since it has been noted that providing information for public engagement on a controversial topic could result in bias ( O’Doherty *et al*., 2012), the background material we provided was designed with this awareness in mind.

Purposive sampling criteria was followed to ensure diversity. The meeting began with two presentations on the material sent out as background reading, followed by a session of questions to provide clarifications where necessary. Deliberation took place in two small groups, of 6–8 participants in each group, over the course of 30 minutes. Questions for deliberation were not sent out prior to the meeting as it could skew discussion ( O’Doherty *et al*., 2012). Three broad questions were provided to groups at the start of deliberation with a moderator facilitating discussions. A short period of individual reflection before deliberation was provided. Of the three questions provided, one pertained to views on GE while the other questions covered perceptions, values, beliefs and expectations in relation to biobanking and genetic research. Suggestions for appropriate procedures to ensure that people’s voices were incorporated as best practice for such research were also elicited. At the conclusion of small group deliberations, one member from each group presented a summary of their discussion. A consensus building poll was conducted to gauge perspectives on the points shared. While the topics for voting were established at the end of the meeting, polling took place by means of a Google form emailed to participants. The audio recording of the deliberations was transcribed and thematically analyzed along with the participants’ individual notes and Google form data.

Participants expressed both positive expectations as well as clear concerns. GE was considered beneficial if it could: provide cures for genetic disorders; had therapeutic value and; improved health and quality of life. It was expected to have social value and be used for “ *the greater good*”. A concern expressed was the misuse of such technology, particularly “ *by the rich*” for the “ *modification of appearances*” and for the creation of “ *designer babies*”, as well as the impact on inheritable traits. There was a fear expressed by participants that exploitation of GE by companies for “ *commercial use*” was a possibility. “ *Who will regulate*?” was a theme revealed as was “ *the unknown long-term impacts”* on society.

Suggestions for more robust governance including a framework which would ensure societal value, control profiteering and prohibit decision-making based on individual demands and interests. Strict ethical guidelines with punitive action for ‘wrong doers’ was also mentioned. The next phase of research will seek a more focused engagement with key stakeholders and those identified as key influencers – each having different interests and needs. Table 1 below provides a description of groups we have considered as stakeholders and influencers.

**Table 1.** Stakeholders and influencers identified during the research process.

Identified groups for engagement	
**Key stakeholders**	Researchers and scientists engaged in genomic research contexts
	Regulators at state, national and organizational levels
	Doctors and clinicians involved in genetic testing and disorders
	Private sector organisations involved with genetic testing and biobanking
	Patients and patient support groups of rare diseases and genetic disorders
**Potential influencers**	Media personnel and communication specialists
	Legal practitioners
	Religious group representatives
	University students
	Information technology professionals and data scientists
	Academics and university faculty
	Non-government organization activists

The Indian community of biotechnologists and medical scientists do not want to be left behind in global advancements of GE and clinical trials to address medical conditions. Hence, given the advancements in medical science and the nascency of regulations and laws, understanding the views of Indian publics is critical. Our research suggests that structured, facilitated multi-stakeholder engagement is required to take place to better understand people’s perceptions, expectations and concerns about GE and to negotiate boundaries and develop appropriate guidelines.

## Reflections on Case 2

The ethical issues that relate to public engagement and social acceptance of genomic research, genetic testing and GE are complex. While the benefits of these technologies are broadly appreciated, there are concerns at two broad levels.

*Social Justice*. Will the benefits of genomic research and GE reach everyone and will it ensure societal value and common good for publics? There is a risk that the utilitarian view of ‘the greatest good for the greatest number’ will prevail at the expense of the voiceless and already marginalized and stigmatized in society. India, with its socio-cultural prejudice against dark skin, short stature, and female children, could see new technologies in genetic manipulation advance mindsets of eugenics and aggravate pre-existing stereotyping, injustices and inequalities.

*Trustworthiness of health systems and regulators*. Given the lack of confidence and mistrust in the country’s health systems and regulatory processes, questions of who will regulate and how will regulations be enforced are of ethical concern. Without the ethical goals of public engagement, and the pursuit of people-centred, bottom-up mechanisms of governance, safeguards to hold researchers accountable to fair research practices will be weak.

It is an ethical imperative to continue to engage with publics and diverse stakeholders to arrive at appropriate guidelines and best practices which fosters sustained community involvement. Alongside engagement practitioners and science communicators, contributions from experts in the social sciences and humanities are ideally placed to engage diverse stakeholders and publics on issues of most concern to them.

These research findings, along with the work of many other research groups, rely heavily on policy makers to incorporate the evidence generated before the impact of science can be properly realized. This is often achieved through effective partnership between science and government. While collaborative partnerships between research organizations and government decision-makers is not common practice in policy-making, there is an ongoing role for both science and government to find more innovative ways to ensure the findings of research are sufficiently reflected in policy decisions.

## Case 3: Co-developing a community-wide acceptance model for vector control in Burkina Faso

Target Malaria is a not-for-profit research consortium developing an innovative vector control against malaria ( Burt *et al*., 2018). This consortium has partners from three continents: Africa, Europe and North America and its aim is to release, with appropriate regulatory approval, genetically modified mosquitoes containing a gene drive mechanism, that will ultimately reduce the malaria vector population. The project uses genome editing technology to create gene drives, which cause specific traits to be inherited at greater levels than normal so that they can spread throughout populations of mosquito species While significant progress has been made in the laboratory ( Kyrou *et al*., 2018), there is still a long way to go before field evaluation of this technology can be allowed and even longer before its ultimate deployment as a complementary tool for malaria elimination can be considered. This case study describes a community-wide acceptance model for vector control and identifies the multiple challenges and opportunities moving forward.

In 2016, Burkina Faso’s resident population was estimated at 19 million ( Institut National de la Statistique et de la Démographie (INSD), 2018) and its population growth rate estimated at 3.1%. Children under 5 represent 18% of the population (as indicated by 2011–2020 population projections for Burkina Faso's health regions and districts, August 2009, INSD). Notwithstanding all combined strategies and interventions deployed for malaria control on the ground, malaria remains a major public health concern in particular in the Haut Bassins region where the project works and where malaria case incidence in 2018 was estimated at 474 per 1,000 residents.

As part of the program’s step-by-step approach to research and development, a small-scale release of sterile male mosquitoes, which did not contain a gene drive mechanism, but which were genetically modified ( Windbichler *et al*., 2008), was proposed in a village of Burkina Faso. The primary objectives were to understand how a genetic modification would behave in mosquito populations and build trust and engagement with the local communities and key stakeholders.

As the strain of mosquito under study is classified as a living modified organism according the Cartagena Protocol on Biosafety, it is regulated as such by national authorities according to the law 005-2006/AN of 4 ^th^ May 2006 ( Burkina Faso, 2006). An application to carry out a small-scale release was made to the national competent authorities in Burkina Faso providing data for the authorities to carry out their own risk assessment and analysis. A public consultation was also implemented by the National Biosafety Authority as part of its legally required process ( Burkina Faso, 2006).

For a project like Target Malaria, the question of appropriate level of community acceptance to proceed to small-scale release is a very central ethical issue. Existing guidelines about the scope and extent of community acceptance is currently limited to analysis confirming that individual informed consent is not an appropriate mechanism for establishing community acceptance ( WHO/TDR & FNIH, 2021). According to the definition proposed by McRae *et al*. (2011) in public health, individual informed consent is required when an individual has direct interaction with investigators, is “directly intervened upon by an investigator”, or their identifiable private data is used ( McRae *et al*., 2011). In the case of the release of male mosquitoes into the environment for vector control, the criteria defined by McRae are not fulfilled (unless the experiment is requiring blood samples) and thus there are no human subjects ( Kolopack & Lavery, 2017). Informed consent is also more relevant for individual decision-making and these releases are taking place in collective spaces and not affecting individuals directly.

The second edition of World Health Organization’s *Guidance Framework for Testing Genetically Modified Mosquitoes* reiterates that individual consent should only be required if specific data is collected from human subjects (such as blood samples for epidemiology study), while the appropriate consent for the release of genetically modified mosquitoes should be obtained at the community level ( WHO/TDR & FNIH, 2021). Similarly, the National Academics of Sciences Engineering and Medicine (NASEM) report on gene drive organisms did not provide any specific comment on a consent model for establishing community acceptance. It instead highlighted the importance of a meaningful engagement reaching beyond a deficit model of engagement where acceptance is only driven by the provision of sufficient scientific information ( NASEM, 2016). Existing international guidance on living modified organisms (for instance the Cartagena Protocol) offers little additional (practical) advice for researchers on how such community acceptance should be obtained, measured and recorded.

The Target Malaria team has consulted other projects in the search for innovative vector control approaches, in particular the model implemented by Eliminate Dengue (now the World Mosquito Program) ( Kolopack *et al*., 2015). This model has helped developed the framework for Target Malaria’s model, but this model was further adapted taking into consideration the perspectives from the communities where the project intervenes. In alignment with its value of community co-development, the Target Malaria project decided to co-develop its engagement model directly with affected communities applying their own knowledge and experience in responding to the question of what constitutes fair and legitimate authorization for field studies of genetically modified mosquitoes and ultimately gene drive mosquitoes ( Hartley *et al*., 2019). As the model continues to be implemented, reflection on the co-development process and the challenges raised continues to be discussed with external parties to strengthen the model.

### The co-development of the community acceptance model

An iterative dialogue between researchers and community members resulted in the initial design of the engagement model. The process involved several phases including: the conception of the design of the model; the selection of community representatives who could speak on behalf of the community and; traceability of the agreement. The model was designed on the basis of what was acceptable to the community that would be directly affected by release of the genetically modified strain of mosquito. Through this process, different governance mechanisms have been put in place with stakeholders attempting to address several challenges – for example, around research monitoring, and national level engagement. Ethical questions raised by the balancing of existing community governance mechanisms with at times conflicting ethical considerations – such as gender and minority representations – has challenged the process and led to significant learning along the way. Despite the implementation of the co-development and acceptance model, external challenges about the legitimacy continue to be raised. A few civil society organizations have questioned the legitimacy of the decision-making committee that had been nominated by the community to express the decision on its behalf and inquiring about whether an appropriate level of information had been reached before the decision was taken (ETC Group 2019). This was further discussed with the community to ensure that the project had the appropriate level of acceptance, and they confirmed, to the project, to the National Biosafety Agency representatives during public consultation, and to some journalists that they agreed with the release. The release took place on 1 ^st^ July 2019 in the presence of village representatives as well as the monitoring group appointed by the community to monitor the process and report to the rest of the community.

This process of co-developing the community acceptance model and ensuring that it is legitimate amongst the different groups that compose the community is a lengthy one. It started while the project was still working on mosquito population characterization through mosquito collections several years before the release itself. As such the process was quite expensive as it required the investment of several researchers and engagement practitioners to develop this framework and implement it. Between 2016 and 2020, the engagement budget in Burkina Faso represented about 23% of the country’s total project budget. As the project moves to its next steps, it is expected that on one side some economies of scale can be made as well as the benefit of having laid some ground principles and work, but on the other side the expectations for engaging a broader group of stakeholders will also require more resources.

A dilemma presents itself where the legitimacy of a co-development process between the project proponents and an affected community is not easily reconciled with values introduced by critical external stakeholders who are not directly affected by the activity. The authors contend a collaborative and reflexive process which advances international guidelines for establishing appropriate acceptance models while ensuring the values of co-development remain is essential.

## Critical Reflections on Case 3

This case highlights the complexities raised in balancing culturally appropriate models of acceptance co-developed with affected communities and those broader ethical considerations that are considered rights-based and regarded as universal. This is not a new debate in research bioethics ( Chin, 2015).

The absence of clear guidelines places the burden of responsibility on researchers, who not only have to apply a clearly defined ethical framework but also must participate in the establishment of this ethical framework. This raises a number of challenges.

The first is one of human resources and competence. Asking researchers to fill the gaps of existing guidelines presumes that research projects are equipped with staff who have adequate training, experience and time to reflect on the range of ethical and practical options for acceptance models. While public health projects are increasingly transdisciplinary ( Hadorn *et al*., 2008), not all research projects in early phases of discovery have the capacity to develop such a framework.

The second challenge concerns questions of legitimacy and societal trust. In the absence of clear guidelines, researchers are looking at a broad spectrum of literature and experiences to establish a workable model. However, the legitimacy of the model designed remains highly dependent on the trust that stakeholders and society place in these researchers. The co-development model is a way to build this legitimacy by sharing the responsibility and trust with the affected community who is expressing what a good model for them might be and together with researchers finding a model that can satisfy their cultural perspectives as well as ethical principles.

The third challenge is one of scalability. Research projects can develop a workable model for the communities where they operate according to the broad ethical guidelines available and fill the gaps in the guidance by working with communities to find what is acceptable. This model relies heavily on a process of identification and analysis of social mechanisms in order to assess the legitimacy and representativeness within the community. However, this model is hard to scale as it relies on intense engagement with stakeholders (e.g. in the case of the village for the small-scale GM mosquito release study it took over six months to co-develop the acceptance model with the community). Legitimacy is built through direct dialogue and thus it is labor-intensive.

Finally, there is an issue of potential perception of conflict of interest, where the project is carrying out both the information and the consultation activities. One could be concerned that a project could hide potential risk or inflate potential benefits in order to get an activity accepted. At the same time, it is crucial for those engagement activities to be carried-out by the activity proponent because this is the basis for trust building and for accountability. The supervision of those activities by institutional ethics committee is a safeguard for this potential risk – very much like in clinical research – but might need to be further strengthened for instance by sharing the information about the IRB process with the public at large.

Absence of clear guidelines is forcing researchers to assume different responsibilities and roles. This might not be possible by all research projects and requires resourcing, additional skill and funding commitment. However, a co-development model reveals the importance of a culturally appropriate approach and would advocate for national or regional guidelines to maintain the balance between universal rights-based approach and a more culturally appropriate approach.

To conclude, for guidelines to be effective across the multiple and varied sectors that are concerned with area-wide public health interventions (as well as land management and conservation communities) cross-sectoral dialogue focusing on key bioethical considerations is required. Such dialogue would require acknowledging the different values and creating a framework to address those diverging views. A common ethical framework for such interventions is now lagging behind the technological innovations available. 

## Discussion and conclusion

The three cases presented here expose the multiple and interrelated challenges, tensions and goals which the spectrum of communication and engagement in the development and use of genome editing for human benefit present. These cases highlight the cultural, institutional and ethical considerations that engagement science and practice demand. Engagement is not a uniformly structured process able to be transferred from one context to another, nor is it the responsibility of a single group or sector. Several important themes emerge from these case presentations.

First, engagement with communities and publics requires clarity of purpose and intention. Engagement for its own sake, or as a demonstration of compliance with organizational guidelines is at best ineffective and at worst erodes trust between science and the beneficiaries of science. While biotechnical scientists may feel a burden to lead engagement initiatives, there are highly skilled professionals who may be better placed to design and conduct engagement activities. The role of engagement practitioners, facilitation professionals, knowledge brokers and science communicators in engagement initiatives could be further explored as professionals who are likely to possess the necessary skill and independence to engage effectively. The contribution of the social sciences to engagement science and practice should not be underestimated. As Cases 2 and 3 highlight, acknowledging multiple values among diverse stakeholder groups is a critical step in building relationships. The governance of engagement *processes and practices* is an implicit yet often overlooked component of the governance of responsible science. 

Related to clarity of purpose, is the need to acknowledge the covert influence of power in research partnerships and funding models which can negatively influence the design, timing and conduct of engagement outcomes. Tensions such as conflicting organizational mandates, donor expectations and resource pressures often impact significantly on the quality and direction of engagement initiatives which have the potential to compromise original intent to work differently. Exercising transparency on the part of innovation advocates, investors and science developers, as was envisioned in Case 1, can similarly shape the process of interaction where building trust between science, government and communities is a longer-term goal.

Quality engagement processes take time to scope, design and implement. For more deliberative processes, monitoring and evaluating the impact of engagement activities at various timepoints may assist practitioners in adapting to changing stakeholder needs. Importantly, establishing broadly shared goals at the outset of engagement initiatives will enable participating groups to assess when these goals have been achieved.

The cases highlight the importance of having social scientists, communication specialists and stakeholder engagement practitioners to support research projects and dialogue efforts with populations participating or impacted by the research. In all three cases, these experts’ knowledge and skills have been critical to the process, both to ensure appropriate design and implementation, but also to be able to reflect on the process and identify learnings that could help future projects. This importance is increasingly recognized in the literature (cf. Lowe *et al*., 2013). Yet, there is a scarcity of human resources and trained staff to carry out such work. This has been highlighted before but remains unaddressed ( Wight, 2008). A broader reflection is needed to integrate these much-needed skills into curriculums.

Finally, with the re-emergence of global responsible science and innovation agendas, there are myriad conceptual engagement frameworks available which draw from other contexts including national resource management and biosecurity ( Warner, 2012), mining ( Delborne *et al*., 2020) and nanotechnology ( PytlikZillig *et al*., 2018). While learning from the lessons of others has its merits, the research community should resist automatically transposing one setting for another without careful consideration of origin, intent and purpose. As is revealed in Case 3, models of consent may guide overall vision, but their co-development is key to ensure that they remain adequate for the specific context.

## Author contributions

The first four authors contributed equally to drafting individual Case Studies and participating in multiple rounds of revision to consolidate Case Studies into a single paper. The final author was responsible for crafting the abstract, background, discussion and conclusion sections and editing the Case Studies for publication.

## Notes

^1^ https://www.iica.int/en/press/news/argentina-presents-international-statement-agricultural-applications-precision Accessed 12 August 2020

^2^The event is available for viewing here https://www.youtube.com/playlist?list=PL9T-lMu3vHFK3N2EIYEscwvttHYEaZ3MK Accessed 10 July 2020

^3^CRISPR, short for clustered regularly interspaced short palindromic repeats, is a gene editing technique that can be used to modify the genome of a living organism.

^4^The multiple news stories generated from this event are available here, accessed 10 July 2020. https://cutt.ly/RoflGtM; https://cutt.ly/3oflMG6; https://cutt.ly/wofl6Ey; https://cutt.ly/Tofzpld

^5^For links to the Twitter radio show, visit https://twitter.com/NacionalAM870/status/1121855165424914434?s=20

https://twitter.com/NacionalAM870/status/1072926267551481857?s=20 Accessed 10 July 2020.

^6^A scenario-based variant of the case study method.

^7^This is a fellowship from the Global Forum for Bioethics in Research, titled ‘Developing a people-centred governance mechanism for biobanks and genetic research with stored samples and data in India.’, the investigators of whom are : Manjulika Vaz, Mario Vaz, Susan Bull and Calvin Wai-Loon Ho.
