# Supplementary material for: Engagement and social acceptance in genome editing for human benefit: Reflections on research and practice in a global context
Source: Wellcome Open Res. 2021 Jul 2;5:244. Originally published 2020 Oct 16. [Version 2] doi: 10.12688/wellcomeopenres.16260.2 (PMC8142603; doi:10.12688/wellcomeopenres.16260.2)
Supplement: Peer review history

# Peer review: Reviewer response for version 1

**DOI:** 10.21956/wellcomeopenres.17863.r43569

**Published:** 2021-05-24

**Authors:** Peter Mills

**Affiliations:** 1 Nuffield Council on Bioethics, London, UK

This open letter offers three, diverse case studies of public engagement on the theme of genome editing to illustrate some of the challenges and share some insights into undertaking engagement as an element of responsible research and innovation. The focus is on governance of engagement (broadly defined).

 The case studies are nicely presented, albeit with slightly uneven balance between the theoretical reflection and empirical description (for example, there is quite a lot of introductory orientation in the second study which might have been considered in the general introduction -- see below -- or perhaps set aside to include in a longer treatment). In fact, if I have one general hesitation, it is over whether what can be gained -- specifically about governance of engagement -- from presenting three very diverse studies in a single open letter outweighs what is lost by stunting what might have been three more detailed and fascinating papers in order to fit the format, particularly when the conclusion suggests (rightly, I believe) such a high level of context-relativity. The thread of ‘genome editing’ that purports to hold these studies together — different in context, as the authors say, but also in purpose, scope and mode, as they are — does not bear very much plucking. If this is a problem, however, it is mostly a problem with the title -- which suggests that the 'genome editing' that is in view in each case is a coherent thing that has enough conceptual gravity to build the paper around.

 If one is to read the paper in the way suggested -- i.e. as offering insight into governance of public engagement -- it might be helpful if the authors distinguished more clearly between design challenges, management of process and governance. There were places where I did not feel that these differences were handled with sufficient sensitivity or the governance insights were drawn out, and there is a tendency to say more about the potential of engagement in the governance of technologies than about the governance of engagement itself. Read in this second way, however, the paper offers some points of great interest.

 I have just a few more specific observations on the sections of the paper.

**Case 1 (Argentina)**

 This describes what is in effect an ‘outreach’ event, effectively to publicise the national commitment to genome editing technologies (rather than any specific application).

 I am slightly worried by the description of the process "taking care to build public trust in the science and governance of GE technologies", not because it seems highly instrumentalising (which it may well have been) but because it is presented in the paper without any critical comment. 

 The goal of this is described as being to ‘empower the audience by providing... information’. Could more be said about how it was (meant to be/succeeded in being) empowering? (Otherwise, impressive coverage might seem a little thin as an evaluative criterion.) 

 The 'reflections' at the end of the case seem to offer mostly operational recommendations but it would be good to hear *how *the 'communication design aspects... retained accountability, trust and transparency'. 

**Case 2 (India)**

 It seems odd that a canonical characterisation of public engagement (referencing Goisauf & Durnová, 2018) comes mid-way through the second case presentation rather than in the general introduction (NB link missing). And it seems that the the first case (the Argentine example) does not easily fit the description.

 There follows a really interesting and well presented description of the initiative, though the 'reflections' could provide more of a reflection on the example itself -- I didn't feel that this linked back as strongly as it could to the process that the authors had taken the trouble to describe so well. 

**Case 3 (Burkina Faso)**

 This case study raises really very profound questions about legitimacy, and the role of engagement alongside domestic political processes and external research governance (albeit largely untried). It outlines an iterative, collaborative and reflexive process as a solution to this. Quite a lot of space is given to explaining the objectives of Target Malaria compared to the public engagement specifically - it is very good stuff but could it be reduced to maintain the focus of the section and the balance with other sections?

 The sentence “unless specific data is collected from human subjects (such as blood samples for epidemiology study), the appropriate consent should be obtained at the community level” might be revised — surely the individual consent would be in addition to the community consent, not an alternative to it. 

 The section on developing community acceptance is very important (although I'm surprised that it is described as 'acceptance', as it recalls the deficit model (which has already been dismissed). 

 I would also be interested in any reflections here on about the definition of 'community' ,which is very important in this case, and it is good to highlight community practices and structures of power from an anthropological perspective (particularly in relation to the National Biosafety Agency and the 'journalists' with whom they interacted). This seems important to defend against the challenges of external stakeholders noted.

**Discussion**

 Three very clearly stated and important points about the role of engagement in governance of research and innovation are made here that, while perhaps not novel, are valuably exemplified in the foregoing studies. 

 Aside from these comments I noted just a few very minor infelicities that should be picked up by another proof read:

 ‘important insights about the practice of engagement is’

 ‘unscrupulous individuals’ intent on achieving’ (NB break this long sentence)

 A misplaced reference in first sentence of Case study 3 and some missing punctuation. 

 Thank you for the opportunity to review this very interesting collection of reports and reflections on three diverse initiatives, which I think are a valuable contribution to understanding the value and practice of engagement. I am content to approve this, although I recommend that some attention is given to the issues I have mentioned above before the paper reaches its final state.

Does the article adequately reference differing views and opinions?

Partly

Are all factual statements correct, and are statements and arguments made adequately supported by citations?

Yes

Is the Open Letter written in accessible language?

Yes

Where applicable, are recommendations and next steps explained clearly for others to follow?

Yes

Is the rationale for the Open Letter provided in sufficient detail?

Yes

Reviewer Expertise:

Bioethics, public discourse ethics, regulation, governance of science and technology.

I confirm that I have read this submission and believe that I have an appropriate level of expertise to confirm that it is of an acceptable scientific standard.

# Peer review: Reviewer response for version 1

**DOI:** 10.21956/wellcomeopenres.17863.r43564

**Published:** 2021-05-24

**Authors:** Maneesha Inamdar

**Affiliations:** 1 Jawaharlal Nehru Centre for Advanced Scientific Research, Bengaluru, Karnataka, India

The report highlights the significant differences and challenges in public engagement practices for gene editing through three different scenarios in diverse contexts. Case 1 describes successes of the Argentine government-backed scientists and policy makers to engage with the public on gene editing. Case 2 describes methods to gather views of diverse stakeholders in India on gene editing. Case 3 assesses the Target Malaria program in Burkina Faso and highlights discordance with existing international guidelines in ensuring local community engagement and involvement in decision making. The authors aim to suggest paths to achieve broad societal involvement at every step of GE, through their work. Most reports on effective public engagement in GE emerge from parts of the world that have advanced scientific and technical expertise in this area and often well-defined policy and/or legislation. However as most countries that may be targets or beneficiaries of GE lack access to this expertise, this article is timely and important in highlighting local contexts that are more likely to apply to their respective parts of the world. Lessons learned from these could be generally applicable in a wider context.

 However, whilst the manuscript draws attention to a number of key points made by describing the challenges and lessons learnt in different contexts, these could be better connected. As the authors aim to present how engagement is conceived, planned and implemented, there could be more synergy between the commentaries. It would help to try and synthesize what “works” in spite of the differences in context. This would be a useful template of minimal points of action for other countries trying to build their engagement frameworks. The authors could add significant value to the manuscript by suggesting how the challenges may be overcome and recommending processes and players that might be taken forward regardless of context, so as to create effective engagement mechanisms or activities.

 Some specific comments:

 In all cases it would be helpful to have quantitative data, which may give some sense of how comparable the scenarios are. For example, in case 1, where the event was transmitted by Twitter- it would be useful to know what percentage of the population has access to this. Such data could help determine applicability to other contexts.

 In case 1, the presenters used visual cues such as images instead of text where possible. This makes the engagement language–independent and is a powerful tool easily applicable in diverse contexts. The authors reflect on Case 1 that there was no precedence for organizing a GE engagement event involving multiple government agencies. They could highlight the advantages of this situation as it behooves organizers to innovate, take responsibility and establish locally relevant standards and solutions.

 Recommendations made at the end of Case 1 are reasonable and sensible and should be applicable in most contexts. It would be helpful to state which recommendations are generally applicable.

 Case 2, makes the important point that ethical and social considerations find limited mention in some of the gene therapy related guidelines in the Indian context. While the comparison to GM crops is useful in explaining how regulation of GE may be circumvented, the authors could highlight the special social and ethical challenges that GE presents and how building consensus in such a diverse and pluralistic society as India is a major task - here too, conveying the extent of diversity through numbers would be useful.

 The authors state that journalists have appealed for more dialogue with the public on GE- it is not clear who this appeal has been made to- especially as journalists are closely engaged with the public. The authors could add clarity to this point. The suggestion of people centric government framework could be qualified by giving actual recommendations or the path forward.

 In the approach in case 2 quantitative information is essential. In Table 1- how many members per group, how was group size determined, was there overlap among groups? What percent of representation of each expertise? How was diversity of representation assured? Were social scientists, ethicists, policy makers, authors of popular science and science fiction included?

 The reflections of Case 2 make important points about social justice and highlight concerns about GE not reaching the underrepresented while, aggravating stereotypes and inequalities. However the section on the role of regulators could be rephrased- it gives the impression that by default scientists may resort to unfair research practices.

 Case 3 describes the experience of Target Malaria team in co-developing an engagement model with researchers and the community. This is no doubt an important path though it can present many additional challenges. Mainly the legitimacy of external agencies with a stake in the gene drive program but not directly affected by it and hence by any negative outcomes of the gene drive. The importance of locally developed context appropriate guidelines involving the community, while taking cognizance of international methods, is emphasized here.

 The authors reflect on the significant challenge researchers face when establishing a new technology for application, where they are expected to build and apply ethical frameworks in addition to doing their research. This presents a major issue of lack of critical mass of trained researchers as well as experts on social and ethical aspects of the technology. Further they highlight the resources and additional skills required for this, which are often not appreciated. This is another common concern across the three cases and could be analyzed accordingly and discussed in depth.

 The authors’ observations are based in different contexts and the presentation and output formats differ. By reporting on three different scenarios the authors do bring out the diversity of values and principles and challenges each situation presents. However a synthesis of the common lessons learnt and proposing general principles that arise in spite of the 3 different scenarios would be more helpful. The challenges of resources, expertise, societal trust, legitimacy, scalability are common around the globe. The authors missed the opportunity to give insight into the unique issues and challenges in less advanced countries. The message that comparison of multiple seemingly very different contexts can yield new ideas and mechanisms to achieve engagement goals is missing. Finally, engagement is of limited value without empowerment. The authors could have included these aspects in their discussion and recommendations.

Does the article adequately reference differing views and opinions?

Partly

Are all factual statements correct, and are statements and arguments made adequately supported by citations?

Yes

Is the Open Letter written in accessible language?

Yes

Where applicable, are recommendations and next steps explained clearly for others to follow?

Partly

Is the rationale for the Open Letter provided in sufficient detail?

Yes

Reviewer Expertise:

Stem cell biology, developmental biology, gene editing

I confirm that I have read this submission and believe that I have an appropriate level of expertise to confirm that it is of an acceptable scientific standard.

# Peer review: Reviewer response for version 1

**DOI:** 10.21956/wellcomeopenres.17863.r43568

**Published:** 2021-05-21

**Authors:** Tetsuya Ishii

**Affiliations:** 1 Office of Health and Safety, Hokkaido University, Sapporo, Japan

The present Open Letter introduces the concise case studies of public engagement regarding genome editing in Argentina, India and Burkina Faso. Because the introduction of genome editing into society will progress in each country, the three cases would be instructive to researchers and policymakers in other countries who aim the acceptance of some applications of genome editing. Therefore, it is worth publishing the information provided here.

 The paper lacks the backgrounds of public engagements in biotechnology and necessary engagement for public acceptance. First, consider a conventional biotechnology, genetic modification (GM) technology. GM crops are grown and somewhat accepted in Argentina and India, compared with other countries, such as Japan (GM crop importer, not cultivated here). However, some consumers in those countries do not accept GM crops or food. The non-acceptance has been associated with five issues of science literacy, trust in the government, researchers, and companies, the perception of risks and benefits, and ethics. The authors should put such grounds in older biotechnology. Second, Discussion and Conclusion is somewhat short, requiring more thoughts particularly regarding the timeline of public engagement of genome editing. Now, there are only a few items of genome editing (soy oil in USA, tomato in Japan, etc.) put into society worldwide. In many countries, public engagement requires prior to the social introduction of genome editing. However, is the prior engagement sufficient for public acceptance? The public engagement will be necessary surrounding key events.

 Regarding Table 1, I ask the authors to put priority or significance in key stakeholders and potential influences, if applicable or available. Lastly, please pay attention to syntax: genome editing and gene editing coexist in the text.

Does the article adequately reference differing views and opinions?

Partly

Are all factual statements correct, and are statements and arguments made adequately supported by citations?

Yes

Is the Open Letter written in accessible language?

Yes

Where applicable, are recommendations and next steps explained clearly for others to follow?

Partly

Is the rationale for the Open Letter provided in sufficient detail?

Yes

Reviewer Expertise:

Bioethics

I confirm that I have read this submission and believe that I have an appropriate level of expertise to confirm that it is of an acceptable scientific standard.

# Peer review: Reviewer response for version 1

**DOI:** 10.21956/wellcomeopenres.17863.r40996

**Published:** 2020-11-13

**Authors:** Carolyn P. Neuhaus

**Affiliations:** 1 The Hastings Center, Garrison, NY, USA

This paper reviews three real-world examples of public engagement regarding gene editing. The three “engagement stories” are (1) raising public awareness of gene editing in Argentina; (2) eliciting public views and promoting dialogue using social science in India; and (3) co-developing a community-wide acceptance model for vector control in Burkina-Faso. The differences among the cases permit analyses of the strengths and weaknesses of the approaches surveyed. Of course this is not an exhaustive list of public engagement practices, but as a brief review of what has been done already, this is very helpful. It will help people in the field especially consider various ways in which they might make good on calls for inclusive public engagement that inform responsive and responsible scientific practice. It will also permit critical takes on these existing approaches to engagement to improve future engagement.

 While the presentation of engagement stories is useful, the lessons gleaned repeat what is already known about public engagement about gene editing. Their findings are in line with the already generally-agreed upon acknowledgement that engagement is – and must be - context specific and communication strategies must be tailored to the intended audience. The need for trained professional facilitators is well-documented.

 The cases themselves are interesting. I would have liked the authors to expand a bit on two points: (1) The authors missed an opportunity to incorporate costs into the description. Could this information be added to educate people in the field – especially funders – about what it takes to actually practice public engagement? Very little is known publicly about the cost of public education and engagement efforts. (2) The critical reflections on the cases are not all that critical. For example, Case 1 is more focused on public education than an instance of a “legitimate opportunit[y] for broader societal involvement in the planning, conduct and delivery of responsible science.” It was a rather a way for scientists and regulators to communicate to the public what’s going on.  It is a worthwhile and valuable effort to educate publics on the Argentinian government’s position on gene editing and steps for creating trustworthy regulation and governance of biotechnology. But the public Q+A sessions do not quite meet the bar for having exchanged information; the design of the event centers on one-way communication. Is this enough to achieve the loftier goals of inclusive and legitimate governance? The authors do not grapple with the question. Case 2 does not grapple with the downsides of undertaking public engagement as a social science research project. While the research design is quite thorough and the results are fascinating, the gap between research and policy means that the impact of public engagement depends on authorities’ uptake of social science research findings into policy. The case does not include information about the uptake of the social science research and/or pre-established relationships with relevant governing authorities. Regulators are included as key stakeholders but the description does not go further than that. These differing takes (among others) on the strengths and weaknesses in particular of Case 1 and Case 2 are not addressed. The reflection on Case 3 is more thorough than the reflections on Case 1 or 2.

 I thank the authors for putting these three cases side by side to allow comparisons. The paper itself does not undertake a directly comparative analysis of the cases but subsequent analyses might. This further step in the analysis – a comparative approach of which approach is best for which kind of context and why – would push the conversation even further than the current paper.

Does the article adequately reference differing views and opinions?

Partly

Are all factual statements correct, and are statements and arguments made adequately supported by citations?

Yes

Is the Open Letter written in accessible language?

Yes

Where applicable, are recommendations and next steps explained clearly for others to follow?

Partly

Is the rationale for the Open Letter provided in sufficient detail?

Yes

Reviewer Expertise:

Bioethics, philosophy

I confirm that I have read this submission and believe that I have an appropriate level of expertise to confirm that it is of an acceptable scientific standard.
